# Novel framework for dialogue summarization based on factual-statement fusion and dialogue segmentation

**DOI:** 10.1371/journal.pone.0302104

**Published:** 2024-04-16

**Authors:** Mingkai Zhang, Dan You, Shouguang Wang

**Affiliations:** School of Information and Electronic Engineering(Sussex Artificial Intelligence Institute), Zhejiang Gongshang University, Hangzhou, Zhejiang Province, China; Tongji University, CHINA

## Abstract

The explosive growth of dialogue data has aroused significant interest among scholars in abstractive dialogue summarization. In this paper, we propose a novel sequence-to-sequence framework called DS-SS (Dialogue Summarization with Factual-Statement Fusion and Dialogue Segmentation) for summarizing dialogues. The novelty of the DS-SS framework mainly lies in two aspects: 1) Factual statements are extracted from the source dialogue and combined with the source dialogue to perform the further dialogue encoding; and 2) A dialogue segmenter is trained and used to separate a dialogue to be encoded into several topic-coherent segments. Thanks to these two aspects, the proposed framework may better encode dialogues, thereby generating summaries exhibiting higher factual consistency and informativeness. Experimental results on two large-scale datasets SAMSum and DialogSum demonstrate the superiority of our framework over strong baselines, as evidenced by both automatic evaluation metrics and human evaluation.

## Introduction

With the rapid development of the information society, the explosive growth of dialogue data has attracted researchers to study dialogue systems [1, 2], dialogue summarization [3, 4] and other tasks in the dialogue field. The practical applications of dialogue summarization systems are evident in customer service interactions [5] and doctor-patient interactions [6], highlighting the immense application potential of dialogue summarization. Therefore, the task of converting a large volume of conversational exchanges into a concise, fluent, and readable text, namely *abstractive dialogue summarization*, is becoming increasingly important.

Most existing abstractive summarization models are designed for structured texts such as news reports [7, 8] and scientific publications [9]. With the development of the sequence-to-sequence model [10], the pointer generator [8], and pre-trained models [11, 12], summaries generated for structured texts have been significantly improved in terms of accuracy and readability. However, when it comes to the task of dialogue summarization, these models do not well work due to the unique structure of dialogues.

**Fig 1:**
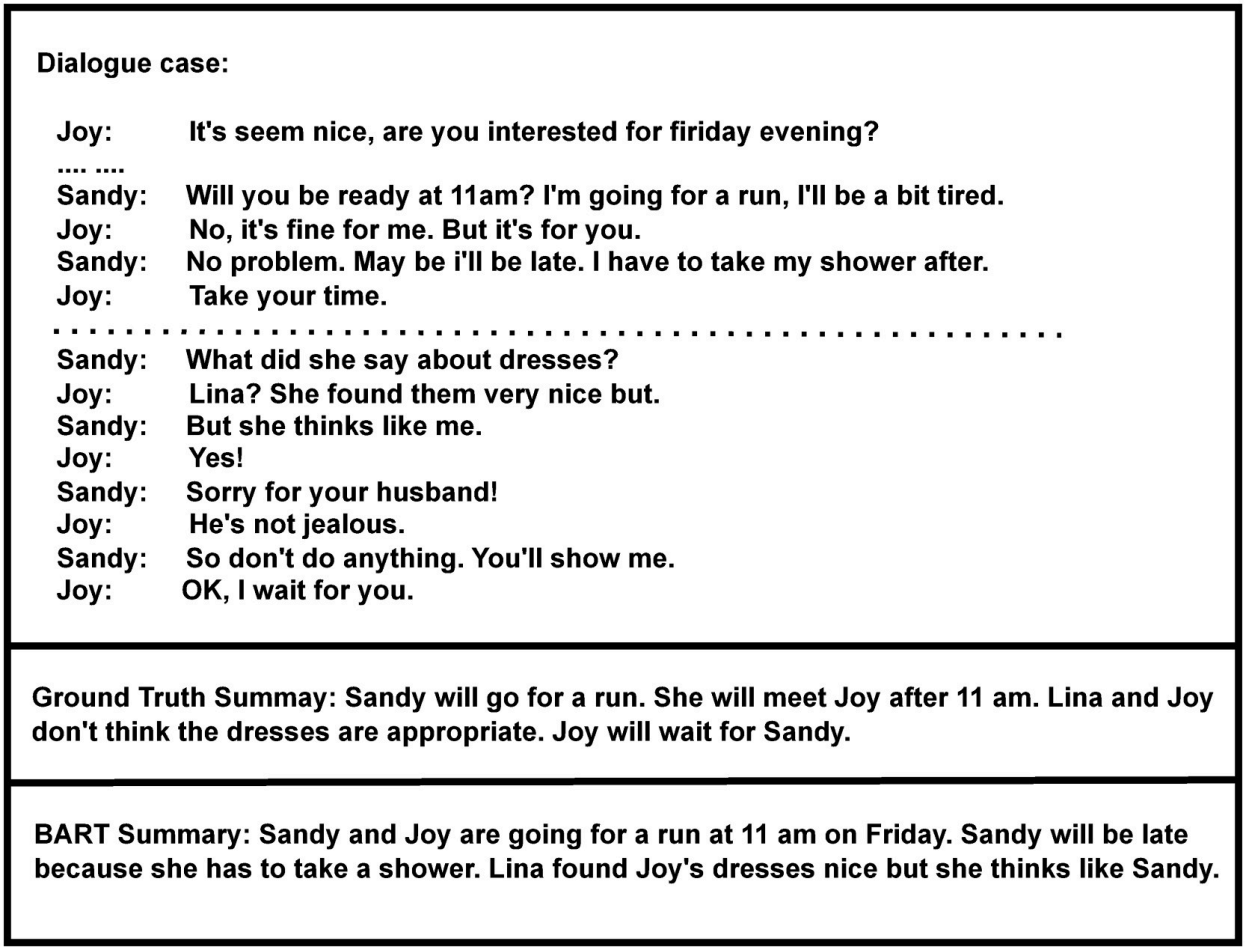
Example dialogue from SamSum dataset [4] with Ground truth summary and a summary from BART [12].

Structured texts typically originate from a single speaker or writer and are expressed from a third-person perspective. In contrast, a dialogue involves two or more participants who express their opinions from their own perspectives. The switching of speaker roles occurs frequently in a dialogue, which brings challenges to the semantic understanding of a summarization model. For instance, in a dialogue example shown in Fig 1, the BART model [12] fails to correctly infer the object “to run” as mentioned in the dialogue, thus leading to a summary content that is not aligned with the ground truth. Additionally, a dialogue involves topic switching and crucial information is often distributed across different parts of a dialogue. Consider again the dialogue in Fig 1. It shows a typical case of topic switching. In more detail, there are two topics: scheduling a time and commenting on a dress. Utterances related to the two different topics are distributed in the upper and lower parts of the conversation. Basically, inputting the entire dialogue directly into a summarization model can hinder its ability to focus on key utterances, potentially resulting in an incomplete summary.

**Fig 2:**
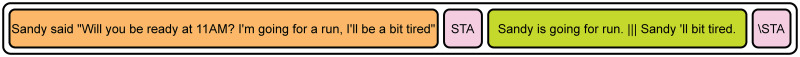
An example of cross-fusing an utterance with its factual statement. <*STA*> and <\*STA*> are special markers.

In this paper, we propose a novel sequence-to-sequence framework, called DS-SS (Dialogue Summarization with Factual-Statement Fusion and Dialogue Segmentation) for abstractive dialogue summarization. We observe that factual triples (<Person, Action, Event>) extracted from a dialogue can effectively describe the progression of dialogue events. Integrating the triple information into the source dialogue might help a summarization model grasp the truth behind events and prevent factual errors in the summary. Therefore, in the DS-SS framework, we generate *factual statements* based on factual triples extracted from a source dialogue and then *cross-fuse* factual statements with the source dialogue, resulting in a *compound dialogue* for further dialogue encoding. Table 1 provides an example of triples and a factual statement generated from an utterance and Fig 2 shows the cross-fusion of an utterance with its factual statement. Compared to the approach in [14] that introduces the triple information into a pre-trained language model through graphical structures, we believe that associating factual statements to their corresponding utterances facilitates a summarization model better identifying the progression of events. On the other hand, inspired by the characteristics of supporting utterance flow introduced in [15], we fine-tune a pre-trained model BERT [11] to separate the compound dialogue into topic-coherent dialogue segments, forming a set of *enhanced dialogues* for dialogue encoding and decoding, which ensures the information completeness of the final generated summary. Experiments are performed on two large-scale dialogue summarization datasets SAMSum [4] and DialogSum [16] to evaluate the proposed framework DS-SS. The results demonstrate that DS-SS outputs a summary with improved performance, exhibiting higher *factual consistency* (i.e., whether the fact in the source dialogue is followed) and *informativeness* (i.e., whether sufficient information in the source dialogue is covered) than baselines.

**Table 1:** Example of factual triples extracted from an utterance using OpenIE [13] and factual statements generated from factual triples.

Utterance	Sandy: I’m going for a run, I’ll be a bit tired.
**Triples**	(Sandy, ’m going for, run)
	(Sandy, ’ll, bit tired)
**Factual Statement**	Sandy is going for run, Sandy will bit tired.

## Related works

### Text summarization

Text summarization has drawn much attention in the area of natural language processing. It can be categorized into extractive summarization and abstractive summarization based on the generation method. Compared to extractive summarization [17, 18], abstractive summarization involves encoding the entire text and generating a summary word by word, which is considered to be a more promising and challenging approach for summarization. Rush *et al.* [10] were the first to apply sequence-to-sequence models to text summarization. To address the out-of-vocabulary problem, See *et al.* [8] introduce a pointer-generator network, enabling the model to copy tokens directly from source documents and use a coverage mechanism to keep track of the words that have already been summarized. Recent research has focused on pre-trained transformer models. Liu *et al.* [11] present a novel document-level encoder based on BERT, showing the process of utilizing pre-trained models for text summarization tasks. Subsequently, Lewis *et al.* [12] introduce the BART model, which makes a significant contribution by incorporating a denoising autoencoder into the pre-training of sequence-to-sequence models. In terms of the ROUGE metric [19], BART outperforms previous methods.

### Dialogue summarization

Dialogues are a special type of texts. Due to the intricate characteristics of dialogues, the general methods for text summarization are often not suitable for summarizing dialogues. Developing specific methods for summarizing dialogues has emerged as a new research field. Dialogue summarization is first introduced by Carletta *et al.* [20] in 2005 for meeting summarization. Gliwa *et al.* [4] in 2019 introduce the first high-quality and human-annotated dialogue summarization dataset, which quickly propelled the development of this research direction. Recent studies have mainly focused on dialogue modeling, often involving additional encoding techniques applied to the text. Wu *et al.* [21] propose a controllable dialogue summary model equipped with a generated sketch. They form sketches based on the intent from the speaker and key information, which serve as weak supervisory signals to fine-tune a pre-trained BART model [12] for generating the final summaries. Feng *et al.* [22] and Kim *et al.* [23] believe that common sense knowledge is the core of dialogue interaction. They introduce common-sense knowledge through knowledge graphs and employ heterogeneous modeling and common sense supervision to improve the quality of generated summaries. Bertsch *et al.* [24] annotate the SAMSum dataset using a perspective transformation approach and improve the zero-shot performance of the data through extraction methods. In this paper, by incorporating factual statements into the source dialogue and segmenting dialogues, we have changed the way in which conversation modeling is approached and enhanced the quality of the final summary.

## Proposed framework

**Fig 3:**
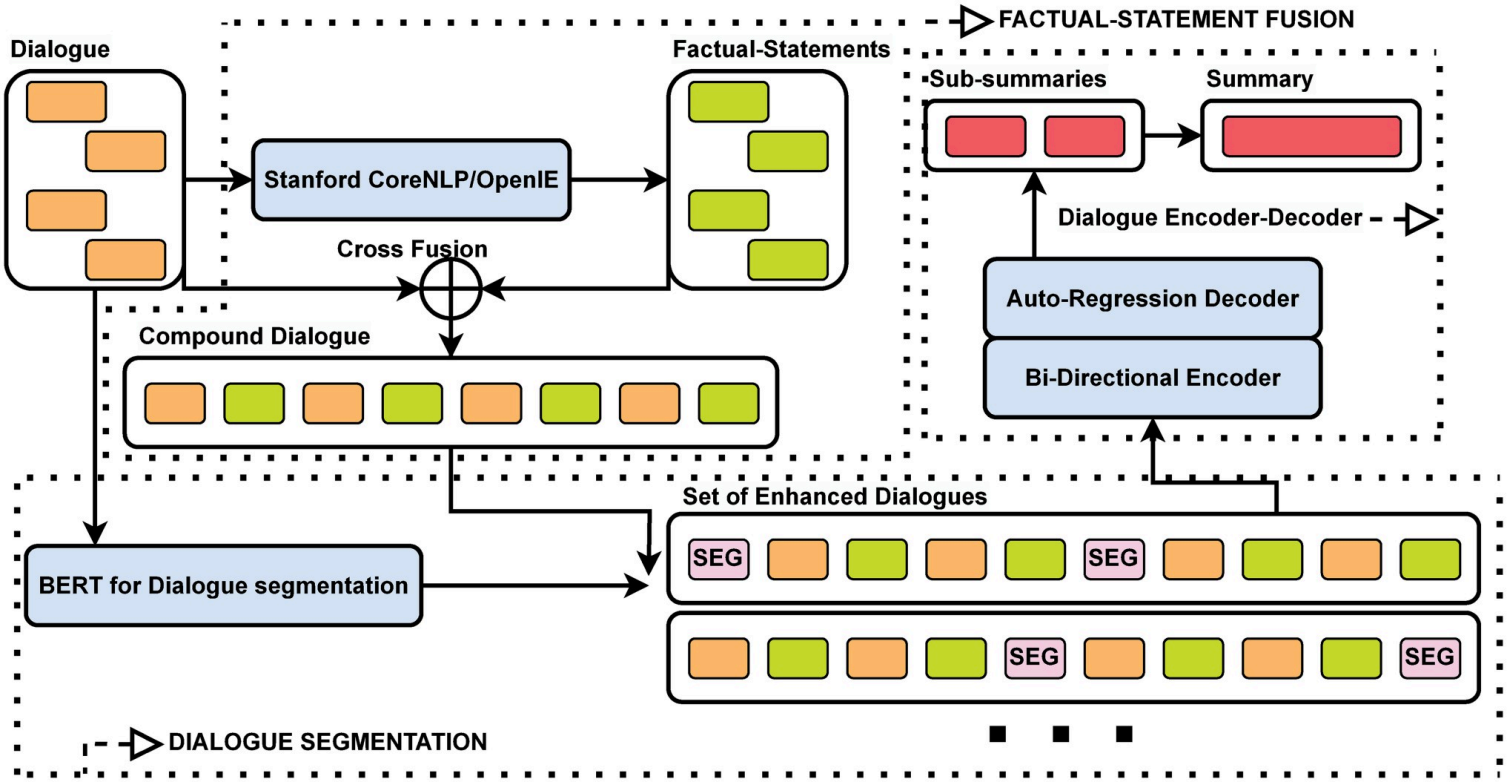
The overall framework of DS-SS. It consists of three modules, namely, factual-statement fusion, dialogue segmentation, and dialogue encoder-decoder. The module of factual-statement fusion is used for incorporating the information of factual triples into the original dialogue, forming a compound dialogue. The module of dialogue segmentation splits the compound dialogue into topic-coherent segments, forming a set of enhanced dialogues. The module of dialogue encoder-decoder generates the final summary by handling the set of enhanced dialogues. (Blue blocks are trainable intermediate components).

In this section, we propose a framework named DS-SS (Dialogue Summarization with Factual-Statement Fusion and Dialogue Segmentation), which is depicted in Fig 3. It mainly consists of three modules, i.e., factual-statement fusion, dialogue segmentation, and dialogue encoder-decoder. Overall, it works as follows: First, factual statements are extracted from the source dialogue and cross-fused with the source dialogue, resulting in a compound dialogue. Second, a dialogue segmenter is trained to separate the compound dialogue into several topic-coherent dialogue segments, forming a set of enhanced dialogues. Finally, by performing the bi-directional encoder and auto-regressive decoder on the set of enhanced dialogues, we derive sub-summaries and then a final summary.

In what follows, we first present the mathematical description of our task and then introduce in detail the modules of factual-statement fusion, dialogue segmentation, and dialogue encoder-decoder.

### Task description

The task of DS-SS follows a sequence-to-sequence problem paradigm. We define the source input as *D* = {*x*_1_, *x*_2_, …*x*_*M*_}, which is a dialogue text consisting of *M* dialogue turns, with each *x*_*i*_ representing a word sequence of a dialogue turn. Our goal is to generate a dialogue summary *Y* = {*y*_1_, *y*_2_, …, *y*_*N*_} consisting of *N* sentences.

To accomplish the task, each module of DS-SS performs its individual sub-task as follows:

- Factual-statement fusion: A set of factual statements *S* = {*s*_1_, *s*_2_, …, *s*_*M*_} is generated based on the source input *D*, where each *s*_*i*_ is a factual statement corresponding to *x*_*i*_ in *D*. Moreover, by cross-fusing *S* and *D*, a compound dialogue D* = {(*x*_1_, *s*_1_), (*x*_2_, *s*_2_), …, (*x*_*M*_, *s*_*M*_)} is generated;
- Dialogue segmentation: The compound dialogue D* = {(*x*_1_, *s*_1_), (*x*_2_, *s*_2_), …, (*x*_*M*_, *s*_*M*_)} is separated into *N* topic-coherent segments, resulting in a set of *N* enhanced dialogues denoted as *D*^#^ = {*X*_1_, *X*_2_, …, *X*_*N*_};
- Dialogue encoder-decoder: By inputting a set of enhanced dialogues *D*^#^ = {*X*_1_, *X*_2_, …, *X*_*N*_} into a dialogue encoder-decoder architecture, the summary *Y* is generated.

### Factual-statement fusion

Neural models designed for summarizing structured texts basically do not work well for summarizing dialogues. Simply inputting a source dialogue into such a neural model often results in a summary with factual errors. One reason we guess is that compared to structured texts like news and scientific papers, dialogues involve more intricate content related to “people, actions, and events”, which is hard to be accurately understood by those neural models. To address this issue, in the proposed framework, we cross-fuse factual statements, which are essentially sentences recording the fact information of “people, actions, and events”, with the corresponding utterances in the source dialogue, forming a compound dialogue to be further handled. This approach aims to enhance the comprehension of the neural model in the event progression throughout the dialogue, ultimately generating a more precise and comprehensive dialogue summary.

Factual statements are derived from triples <Person-Action-Event> extracted from the source dialogue. The process begins by transforming utterances in the dialogue from a first-person perspective to a third-person perspective following specific rules. This transformation involves replacing first/second-person pronouns with the names of the current or surrounding speakers. Additionally, we utilize coreferences clusters in conversations detected by the Stanford CoreNLP [25] to substitute third-person pronouns. Subsequently, we use a well-established Open Information Extraction (OpenIE) [13] to extract triples. The triples extracted by OpenIE have shown useful in downstream NLP tasks as text summarization [26] and question answering [27]. Finally, we join all the items in a factual triple together to generate a clear and well-structured factual statement, where the triples serve as the “subject-verb-object” structure of the sentence.

We note that it could happen that multiple extracted triples reflect the same fact at different levels of granularity [28]. Hence, merging all extracted triples into a dialogue would result in highly redundant data. The redundancy not only increases the computational burden but also confuses the neural model when generating a summary. To address this issue, we adopt a text matching approach to filter duplicate triples. If all words in one triple are covered by another triple, we remove such a covered triple. This filtering mechanism ensures the conciseness of the input data and contributes to reducing the data processing complexity.

Factual statements work as external information to facilitate the neural model in generating summaries. Similar approaches exist in the literature that make use of external information. Most of them employ dual encoders, with one dedicated to encoding the source text and the other dedicated to encoding the external information. Given that factual statements are extracted from their corresponding utterances, it is our contention that inputting the source dialogue and factual statements into separate encoders might result in a partial disconnection of the inherent relationship between the factual statements and their associated sentences. Therefore, we decide to cross-fuse the source dialogue with its corresponding factual statements, forming a compound dialogue to be encoded by a single encoder. Instead of directly appending factual statements to the entire dialogue, the operation of “cross-fusion” involves concatenating each factual statement after the corresponding utterance. Fig 2 provides an example of cross-fusing an utterance with its factual statement. In order to clearly distinguish between the dialogue utterance and the factual statement, we insert special markers <*STA*> <\*STA*>before and after each factual statement.

### Dialogue segmentation

**Fig 4:**
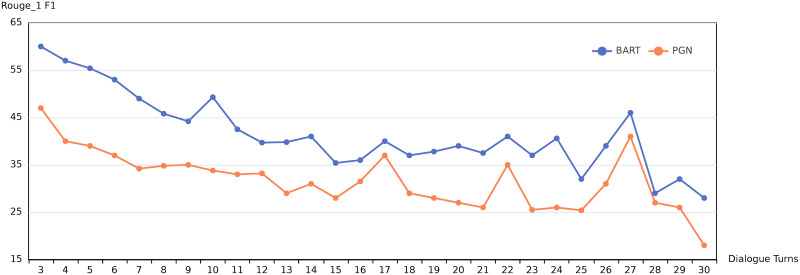
The changing of ROUGE-1 F1 scores as the number of dialogue turns increases.

A dialogue, as a complex form of information exchange, involves topic switching as the conversation progresses. Based on statistics from the SAMSum dataset [4], former summary sentences focus on the former dialogue utterances, while later summary sentences focus on the later dialogue utterances [15]. Furthermore, Fig 4 shows the performance of two summarization models BART [12] and PGN [8] with respect to the number of dialogue turns (from 3 to 30) when summarizing dialogues from the SAMSum dataset. An evident observation is that as the number of dialogue turns increases, the overall performance of the models shows a noticeable decline. Considering the reasons mentioned above, we believe that when generating summaries for dialogues that are topic-coherent and have fewer dialogue turns, the generated summaries can more accurately reflect the content of the dialogue. Therefore, we propose an intuitive solution: we use a pre-trained model to identify suitable segmentation points, separating a dialogue into several topic-coherent segments for summarization.

It is challenging to accurately segment a dialogue into several topic-coherent segments. To overcome this issue, we delve into a solution involving the training of a binary classifier using the pre-trained BERT [29] model to achieve dialogue segmentation. When using BERT for text classification tasks, it is common to use the embedding of the [CLS] token as input for a multilayer perceptron classifier. Our training data is sourced from the dialogue corpus dataset itself. By using Sentence-BERT [30], we compute similarity scores between conversational segments and each summary sentence. Consequently, we select the utterances with the highest score as the segmentation points in our training data. The formula for calculating the segmentation points is as follows:

$$\begin{array}{c} \begin{array}{c} SEG_{i}=argmax_{k}\left[Score\left(X,y_{i}\right)\right],\\ (X=\left[x_{SEG_{i-1}}:x_{k}\right],SEG_{0}=1). \end{array} \end{array}$$ (1)

where *SEG*_*i*_ represents the i-*th* segmentation point and *Score* indicates the similarity score. Based on the segmented data obtain from the dialogue data itself, we can train a model to determine which dialogue turns serve as segmentation points within the dialogue, successfully transforming the task of dialogue segmentation into a binary classification task.

Specifically, we independently encode each dialogue turn and utilize it as an input of the classifier. The input in BERT includes two special tokens, [CLS] and [SEP]: [CLS] signifies the beginning of the input text, while [SEP] marks the junction between two input segments. Considering the study on the impact of BERT input format on text segmentation results in [31], we add [SEP] tokens between each dialogue turns before inputting into BERT. As a result, the input of BERT is changed to the following format:

$$\begin{array}{c} \begin{array}{cc} Input= & \\ \left[[CLS],[SEP\right],x_{1},\left[SEP\right],x_{2},\left[SEP\right] & ,\ldots ,\left[SEP\right],x_{M},\left[SEP\right]]. \end{array} \end{array}$$ (2)

By inputting the organized data, we get the hidden vector *H* from the last layer of BERT, which is the representation of [CLS] token. We use *H* as the input to the linear layer and activation function to obtain the probabilities for segment prediction. The softmax activation function is a commonly used function to transform a set of values into a probability distribution. Through the softmax function, we can obtain the probability distribution of whether dialogue turn is a dialogue segmentation point. The specific formula is as follows:

$$\begin{array}{cc} H= & BERT(Input), \end{array}$$ (3)

$$\begin{array}{c} P=softmax(ReLU(W(H)+B)), \end{array}$$ (4)

where both *W* and *B* are trainable parameters.

The probability *P* is based on the predicted segment probability trained using binary cross-entropy loss. During the training process, we set the learning rate to 1e-5 and the batch size to 8. When employing the trained model for prediction, we set a threshold of 0.5. If the predicted probability *P* exceeds this threshold, it signifies that our binary classifier predicts the current utterance to be a segmentation point. We test the precision and F1 scores of the trained dialogue segmenter on the SAMSum and DialogSum datasets. The results are shown in Table 2. We observe that it achieves the precision of over 87% on either dataset and gets an F1 score of 86.21 on the SAMSum dataset and 87.51 on the DialogSum dataset, which are competitive with results in the field of text segmentation.

**Table 2:** Precision and F1 scores of the trained dialogue segmenter on the SAMSum and the DialogSum test sets.

Dataset	Precision	F1
SAMSum	87.2%	86.21
DialogSum	87.9%	87.51

We treat the portion between the current segmentation point and the previous segmentation point as a topic-coherent dialogue segment. In the case that we can identify *N* topic-coherent segments in the compound dialogue, we may obtain *N* enhanced dialogues, each of which is essentially a compound dialogue with a topic-coherent dialogue segment marked.

### Dialogue encoder-decoder

Our framework, DS-SS, is built upon a transformer-based encoder-decoder architecture. In more detail, we initialize our dialogue encoder with a pre-trained encoder BART-xsum-large [12]. The input to the encoder is the enhanced dialogues. When summarizing an enhanced dialogue, we expect the model to consider the text between two <*SEG*> tags as the core content and combine it with contextual information to generate a sub-summary.

We feed the representation vectors generated by the bi-directional encoder into the decoder, and generate tokens from left to right in an auto-regressive manner by stacking multiple decoders. The training objective is to minimize the negative log-likelihood loss parameterized by *θ*,

$$\begin{array}{c} L=-\sum logP({\tilde{y}}_{l}|y_{1:l-1},X_{i};\theta ), \end{array}$$ (5)

where ${\tilde{y}}_{l}$ is the predicted sequence, *y*_1:*l*−1_ is the first *l* − 1 sequences of the target summary for the current enhanced dialogue.

Finally, after generating multiple sub-summaries separately, we merge them together to form the final summary.

## Experiment setup

### Datasets

Our method is evaluated on two datasets SAMSum [4] and DialogSum [16]. The statistical information of the two datasets is shown in Table 3.

**Table 3:** Statistics of SAMsum and DialogSum, including the total numbers of conversations in the train, valid, test sets, and the average numbers of participants, turns, dialogue tokens, summary tokens.

Dataset	Train	Valid	Test	Participants	Turns	Tokens/dialogue	Tokens/summary
**SAMSum**	14732	818	819	2.4	11.2	82.57	20.30
**DialogSum**	12460	500	500	2.0	9.5	121.56	22.64

The most widely used dataset for the purpose of abstractive dialogue summarization is SAMSum [4]. It comprises 16,369 natural language dialogues created by linguists with manually annotated summaries. To ensure the quality of the data, we undertake specific cleaning procedures, including removing URLs and emoticons from the dialogues.

DialogSum [16] is a recently released dataset. It is the first large-scale scenario-based dialogue dataset collected from real-life conversations. The conversational data in this dataset is sourced from three public dialogue corpora and an English oral practice website. The dialogue summarization task for this database is more challenging owing to the abstractive nature of its summaries.

### Evaluation metrics and baselines

We employ standard ROUGE [32], BERTScore [33] and METEOR [34] as metrics for automatic evaluation on dialogue summarization models. Both ROUGE and BERTScore provide quantitative measures of the similarity between the generated and ground truth summaries, enabling us to effectively evaluate and compare different models. Specifically, the ROUGE metrics include ROUGE-1, ROUGE-2, and ROUGE-L metrics, which respectively compare the 1-gram, 2-gram, and longest common subsequence overlap between the generated summary and the ground truth summary. In the experiment, we utilize the py_ROUGE library with stemming as in the work [4]. In addition, we note that BERTScore is more relevant to the factual consistency of the summaries [35]. We follow https://github.com/Tiiiger/bert_score to calculate BERTScore. Note that different tools may result in different BERTScore. Concerning the METEOR metric, it combines precision and recall, taking into account factors such as vocabulary matching and synonym substitution. The advantage of METEOR as a summary metric lies mainly in its comprehensive consideration of the diversity and accuracy of content, thus enabling a more comprehensive evaluation of the quality of generated summaries.

The following methods are adopted as baselines in our experiment:

- PGN [8]: An RNN-based method designed for text summarization, incorporating a coverage mechanism to address the issue of repeated generation.
- Transformer [36]: A widely used encoder-decoder architecture with self-attention and multi-head attention, serving as the underling structure for most pre-trained models.
- UniLM [37]: A unified language model which is pre-trained using three types of language modeling tasks: unidirectional, bidirectional, and sequence-to-sequence pretrained on English Wikipedia and BookCorpus.
- BART-xsum [12]: A model trained by corrupting text with an arbitrary noising function and learning to reconstruct the original text, fine-tuned from BART using XSUM [38] dataset.
- Multi-View BART [3]: The first dialogue model by incorporating dialogue structure information, introducing topic and stage views on top of BART for dialogue summarization.
- CODS [21]: A controllable dialogue summary model equipped with a generated sketch that is formulated based on intent from speakers and essential information.
- SICK [23]: A model employing an external knowledge model to generate a comprehensive array of commonsense inferences and utilizing a similarity-based selection method to choose the most probable one.

### Implementation details

Our implementation is based on the BART language model from Huggingface [39]. Specifically, we use the BART-xsum-large version for fine-tuning on the dataset. We set the maximum length of the input dialogue to 1024 and the output to 100. The initial learning rate is set to 3e-5, and the training batch size is 16. We apply linear warm-up for the first 600 steps, followed by linear decay, and use the Adam optimizer [40]. All experiments are run on one NVIDIA A40 GPU.

## Experiment results

### Automatic evaluation

We compare the Rouge metrics of our framework DS-SS with baselines using SAMSum and DialogSum datasets. The results are shown in Table 4. We can see that our DS-SS framework outperforms all baselines in terms of all Rouge metrics when applied to DialogSum dataset. As for SAMSum dataset, DS-SS outperforms all baselines in both Rouge_1 and Rouge_L metrics and gets the second highest score in ROUGE_2 metric. The results show the competitive performance of our proposed framework. In addition, we have the following observations on the results in Table 4: 1) Among baselines, pre-trained models (e.g., UniLM [37] and BART [12]) behave better than non-pretrained ones (e.g., PGN [8] and Transformer [36]). It demonstrates the advantage of employing large-scale pre-trained models in downstream tasks like dialogue summarization. 2) All models except PGN [8] achieve higher Rouge scores on SAMsum dataset in comparison to DialogSum dataset. This is potentially because DialogSum dataset contains generally longer conversations with shorter summaries, requiring better abstraction skills for dialogue summarization.

**Table 4:** ROUGE evaluation on the SAMSum and DialogSum test sets. Results with * are obtained from [23],^+^ are obtained from [41].

Model	SAMSum			DilogSum		
	ROUGE_1	ROUGE_2	ROUGE_L	ROUGE_1	ROUGE_2	ROUGE_L
PGN [8]*	32.27	14.42	34.36	33.77	9.24	32.18
Transformer [36]*	42.37	18.44	39.27	35.91	8.74	33.50
UniLM [37]*	47.85	24.23	46.67	42.38	16.88	34.36
BART-xsum [12]	52.37	27.36	48.71	45.11	19.66	39.86
Multi-View BART [3]^+^	49.35	25.61	47.73	42.51	18.93	40.45
CODS [21]*	52.65	27.84	50.79	44.27	17.90	36.98
SICK [23]*	53.39	**28.42**	49.12	46.01	20.30	40.75
DS-SS(Ours)	**53.63**	28.26	**50.87**	**46.36**	**20.33**	**41.07**

### Ablation study

To validate the impact of the factual-statement fusion module and the dialogue segmentation module in our model DS-SS, we conduct an ablation study to compare DS-SS with its ablated versions that we call BART+SEG and BART+STA, respectively. BART+SEG is the one removing the factual-statement fusion module from DS-SS, while BART+STA is the one removing the dialogue segmentation module from DS-SS. In the ablation study, we use datasets of SAMSum and DialogSum, and consider three evaluation metrics, namely, Rouge, BERTScore, and METEOR. The evaluation results are presented in Table 5. We can see that removing either module results in a reduction in scores, but they are still higher than that of the baseline model BART-xsum [12]. This indicates that both modules positively influence the performance of DS-SS model. In more detail, we notice that ROUGE mainly considers the overlap of n-grams, word sequences, and word pair sequences between the generated and the ground truth summaries, thus focusing more on content repetition. BertScore also measures the similarity between the generated and the ground truth summaries but is more related to the factual consistency of summaries. METEOR focuses on both exact matches and semantic relevance, involving not only exact word matching but also synonym substitution, word order changes, and inflectional variations. As shown in Table 5, BART+SEG gets higher ROUGE scores than BART+STA in both datasets while concerning BERTScore and METEOR metrics, BART+STA gets higher scores than BART+SEG in both datasets. Therefore, we infer that the dialogue segmentation module helps achieve better results in terms of text matching, while the factual-statement fusion module contributes more to the improvement of semantic and factual consistency.

**Table 5:** Evaluation on the SAMSum and DialogSum datasets for an ablation study. BART+STA and BART+SEG are ablated models individually removing the dialogue segmentation module and the factual-statement fusion module from DS-SS.

Model	SAMSum			DialogSum		
	ROUGE_1	BERTScore	METEOR	ROUGE_1	BERTScore	METEOR
**BART-xsum [12]**	52.37	68.23	41.03	45.11	70.47	35.09
**BART+STA**	52.89	71.23	42.33	45.72	71.68	36.25
**BART+SEG**	53.45	70.86	42.17	46.02	70.93	35.92
**DS-SS**	**53.63**	**71.35**	**42.65**	**46.36**	**71.72**	**36.83**

### Human evaluation

We conduct a human evaluation to assess the factual consistency and informativeness of the generated summaries, which serve as indicators of the summary quality. We randomly select 50 dialogues from the test sets of SAMSum and DialogSum and provide the ground truth summaries as well as the summaries generated by BART-xsum and DS-SS. We ask 10 evaluators to rate the factual consistency and informativeness of the summaries on a scale from 1 (poorest) to 5 (best). The average scores are presented in Table 6. We can see that a margin exists between the scores for the ground truth summaries and the summaries generated by BART and DS-SS. Meanwhile, DS-SS outperforms the BART model in both factual consistency and informativeness, thereby affirming the capability of the proposed framework DS-SS in reducing factual errors and keeping more complete information in generated summaries.

**Table 6:** Human evaluation results of the summaries from DS-SS, BART, as well as the Ground Truth summary.

	Factual Consistency	Informativeness
**Ground Truth Summary**	4.15	3.96
**BART-xsum**	3.37	3.64
**DS-SS**	3.86	3.81

### Case study

We provide two examples to compare the quality of the summaries from BART model [12] and the proposed model DS-SS. Taking Example 1 in Table 7 as an illustration, we observe that the summary generated by the BART model contains the phrase “She can’t into the salon,” where “She” refers to Gina. However, in the dialogue context, the fact is that Flo cannot go to the salon. Clearly, BART model makes an erroneous coreference resolution. In contrast, the summary generated by DS-SS does not exhibit such a factual error. We believe that our proposed framework avoids such a factual error mainly due to the fusion of the factual statement “Flo can’t get into the salon until the 6th.” In Example 2, BART model overlooks the content in the latter part of the dialogue, that is, “John thinks Igor should do what he has to do,” resulting in a summary with incomplete information. Our model addresses this issue with the help of dialogue segmentation, although it occasionally introduces some redundant information in the summary content. In summary, both examples further validate the advantages of our framework DS-SS in avoiding factual errors and capturing the topic switching in a dialogue, ultimately generating more accurate and comprehensive summaries.

**Table 7:** Two cases for comparing the summaries from DS-SS, BART-xsum, and the Ground Truth summary.

EXAMPLE 1	Dialogue	Flo: OMG, I can’t get into the salon until the 6th!
		Gina: What? Why?
		Flo: They’re just too busy. I’m going to be gray! LOL!
		Gina: Get you a touch-up kit at tesco!
		Flo: Gonna have to.
	**Ground Truth Summary**	Flo cannot get an appointment at the salon until the 6th. Flo worries she’s going to be gray.
		Flo will have to get a touch-up kit at Tesco.
	**BART Summary**	Gina will buy Flo a touch-up kit at Tesco because she can’t get into the salon until the 6th.
	**DS-SS Summary**	Flo can’t go to the hairdresser due to the salon is too busy, Flo has to wait unti the 6th. Gina will
		buy her touch-up kit at Tesco.
**EXAMPLE 2**	**Dialogue**	Igor: Shit, I’ve got so much to do at work and I’m so demotivated.
		John: It’s pretty irresponsible to give theat much work to someone on their notice period.
		Igor: Yeah, Exactly! Should I even care?
		John: It’s up to you, but you know what they say.
		Igor: What do you mean?
		John: Well, they say how you end things shows how you really are.
		Igor: And now how you start, Right?
		John: Gotcha!
		Igor: So what shall I do then?
		John: It’s only two weeks left, so grit your teeth and do what you have to do.
		Igor: Easy to say, hard to perform.
		John: Come on, stop thinking, start doing!
		Igor: That’s so typical of you!
	**Ground Truth Summary**	Igor has a lot of work on his notice period and he feels demotivated. John thinks he should do
		what he has to do nevertheless.
	**BART Summary**	Igor has a lot of work to do at work. He has two weeks left of his notice period.
	**DS-SS Summary**	Igor has a lot of work to do before his notice period ends in two weeks. John advises Igor to do
		what he has to do.

## Conclusion

In this work, we propose a sequence-to-sequence framework called DS-SS for abstractive dialogue summarization. In particular, factual statements are cross-fused into the source dialogue, which assists the basic summarization model in understanding the progression of events in the dialogue. Additionally, a dialogue segmenter is trained to separate a dialogue into topic-coherent segments, which helps improve the informativeness of the generated summary. Experimental results on the SAMSum and DialogSum datasets demonstrate the effectiveness of our proposed framework. Human evaluations further indicate the superiority of the summaries generated by DS-SS in terms of factual consistency and informativeness. For future research, we plan to apply our dialogue summarization approach to practical scenarios such as customer service and medical communication, conducting systematic deployment and evaluation to explore the full potential of dialogue summarization in real-world applications.

## Supporting information

S1 File Datasets used in experiments. (ZIP)

S1 Appendix 1. Automatic evaluation of methods and specific packages used, and 2. Data used to build Fig 4.
(PDF)
