# Supplementary material for: Novel framework for dialogue summarization based on factual-statement fusion and dialogue segmentation
Source: PLoS One. 2024 Apr 16;19(4):e0302104. doi: 10.1371/journal.pone.0302104 (PMC11020369; doi:10.1371/journal.pone.0302104)
Supplement: S1 Appendix — 1. Automatic evaluation of methods and specific packages used, and 2. Data used to build Fig 4. (PDF) [file pone.0302104.s002.pdf]

Support Information

S2 Appendix

1.Automatic evaluation of methods and specific packages used

We employ standard ROUGE, BERTScore and METEOR as metrics for automatic evaluation on models. In the experiment, we utilize the py_ROUGE library (pypi.org/project/pyrouge/) to calculate ROUGE score, follow (github.com/Tiiiger/bert_score) to calculate BERTScore and utilize NLTK package to calculate METEOR score.

2.Data used to build Figure 4

Dialogues Turns	BART	PGN
3	59.9	47
4	57	40
5	55.4	39
6	53	37
7	49	34.2
8	45.8	34.8
9	44.2	35
10	49.3	33.8
11	42.5	33
12	39.7	33.2
13	39.8	29
14	41	31
15	35.4	28
16	36	31.5
17	40	37
18	37	29
19	37.8	28
20	39	27
21	37.5	26

22	41	35
23	37	25.5
24	40.6	26
25	32	25.4
26	39	31
27	46	41
28	29	27
29	32	26
30	28	18
